# Amikacin nebulization for the adjunctive therapy of gram-negative pneumonia in mechanically ventilated patients: a systematic review and meta-analysis of randomized controlled trials

**DOI:** 10.1038/s41598-021-86342-8

**Published:** 2021-03-26

**Authors:** Jun-Ping Qin, Hui-Bin Huang, Hua Zhou, Yuan Zhu, Yuan Xu, Bin Du

**Affiliations:** 1 Department of Critical Care Medicine, Beijing Tsinghua Changgung Hospital, School of Clinical Medicine, Tsinghua University, Beijing, 102218 China; 2 Medical ICU, Peking Union Medical College Hospital, Peking Union Medical College, Chinese Academy of Medical Sciences, 1 Shuai Fu Yuan, Beijing, 100730 China

**Keywords:** Infectious diseases, Drug therapy

## Abstract

Treatment of ventilated patients with gram-negative pneumonia (GNP) is often unsuccessful. We aimed to assess the efficacy and safety of nebulized amikacin (NA) as adjunctive therapy to systemic antibiotics in this patient population. PubMed, Embase, China national knowledge infrastructure, Wanfang, and the Cochrane database were searched for randomized controlled trials (RCTs) investigating the effect of NA as adjunctive therapy in ventilated adult patients with GNP. Heterogeneity was explored using subgroup analysis and sensitivity analysis. The Grading of recommendations assessment, development, and evaluation approach was used to assess the certainty of the evidence. Thirteen RCTs with 1733 adults were included. The pooled results showed NA had better microbiologic eradication (RR = 1.51, 95% CI 1.35 to 1.69, *P* < 0.0001) and improved clinical response (RR = 1.23; 95% CI 1.13 to 1.34; *P* < 0.0001) when compared with control. Meanwhile, overall mortality, pneumonia associated mortality, duration of mechanical ventilation, length of stay in ICU and change of clinical pneumonia infection scores were similar between NA and control groups. Additionally, NA did not add significant nephrotoxicity while could cause more bronchospasm. The use of NA adjunctive to systemic antibiotics therapy showed better benefits in ventilated patients with GNP. More well-designed RCTs are still needed to confirm our results.

## Introduction

Gram‑negative pneumonia (GNP) is a common and serious infection in critically ventilated patients, which accounts for around 65% of pneumonia cases in the intensive care unit (ICU)^1^. It is associated with significant mortality, duration of mechanical ventilation (MV), length of ICU stay, as well as health care costs^2–4^. To date, despite diagnostic and antibiotics improvements, treatment failure for ventilated GNP is not infrequent^5^. Moreover, the presence of GNP caused by drug-resistant pathogens has significantly grown and shown difficult to be eradicated due to the poor lung penetration of intravenous antibiotics, which further complicates the treatment^6,7^. Therefore, therapies that increase local concentration antibiotics in the lung by adding aerosolized antibiotics (i.e., amikacin) have attracted increasing attention^8^.

Theoretically, nebulized amikacin (NA) can be used as an adjunctive therapeutic option in treating ventilated patients with GNP. The advantage to NA in this scenario including achieving high intra-pulmonary concentration that may be effective even for resistant pathogens, thwarting selective pressure and drug-resistant development, and extremely low concentration in the blood due to local administration, thus avoiding dose-dependent systemic toxicity^9–13^.

Although several clinical studies reported the merits of NA in ventilated GNP^12–15^, high-quality evidence to support its use remains limited. Even so, the use of NA in ventilated patients is not unusual. In 2016, a survey of 193 ICUs worldwide showed that NA was prescribed by 27% of the ICUs in clinical practice^16^. Interestingly, the latest American^2^ and European^17^ guidelines for the management of HAP/VAP provided opposed recommendations on the use of aerosolized antibiotics in ventilated GNP. Of note, these weak recommendations are mainly based on observational studies, with very few RCTs focusing on amikacin have been included. Additionally, a recently published meta-analysis in Chinese suggests NA improves clinical response but not mortality rate and other clinical outcomes^18^. However, this meta-analysis mainly included literatures in Chinese. Therefore, the efficacy and safety of NA in such a patient population remain unclear.

Recently, several studies on this topic have been published and some of them have a modest sample size, while the conclusions are inconsistent^14,19,20^. Thus, with the help of the strengthened power of meta-analytic techniques, the present meta-analysis aimed to review the available published RCTs to investigate the efficacy and safety of NA as adjunctive therapy in the treatment of critically ill ventilated patients with GNP.

## Materials and methods

This systematic review and meta-analysis were conducted following the PRISMA guidance (http://www.prisma-statement.org) (Appendix 1). The protocol for this systematic review and meta-analysis was registered on the International Platform of Registered Systematic Review and Meta-analysis Protocols database (INPLASY202070045) and is available in full on the inplasy.com (https://doi.org/10.37766/inplasy2020.7.0045).

### Search strategy

Two authors (H-BH and J-PQ) independently searched the Cochrane Library, PubMed, China national knowledge infrostructure, Wanfang and Embase database for potentially relevant studies from inception to Jun 20, 2021, which is the last search. The details in the literature search terms were summarized in Appendix 2. Our research was limited to RCTs with Chinese and English. Reference lists of relevant studies were also evaluated to ensure that all possible publications were included.

### Study selection

Studies were considered eligible if they met the following criteria: (1) design: RCTs; (2) population: adult (≥ 18 years old) critically ill patients with MV (tracheal intubation or tracheostomy) and diagnosed of GNP (caused by susceptible or resistant pathogens); (3) intervention: patients were randomized to either NA group or control group (aerosolized placebo or no drug), both of which were given alongside intravenous antibiotics during the treatment period (decided by the attending physician based on available culture results or clinical guidelines provided); and (4) predefined outcomes: clinical response, mortality, microbiologic eradication, clinical pulmonary infection score (CPIS), duration of MV and length of stay in ICU. We excluded studies as following: (1) the main focus was children or pregnant women, (2) with any different therapy other than NA between two groups, (3) use of NA as monotherapy, (4) studies focused on in vitro or cystic fibrosis or just pharmacokinetic/pharmacodynamic, (5) available only in abstract form or meeting reports, and 6) studies without reporting predefined treatment outcomes.

### Data extraction and outcomes

Data extraction was undertaken by H-BH and JPQindependently for included studies on study design, patient inclusion criteria, NA and control group regimens, microbiological and clinical cure criteria, as well as predefined outcomes. Authors were contacted where data were unclear or unavailable. The primary outcome was the clinical response (defined as a complete or partial resolution of clinical signs and symptoms of infection, according to the criteria by each study author). Secondary outcomes included overall mortality (defined as ICU or hospital or 28-day mortality, the longest follow-up reported was preferred), pneumonia associated mortality, microbiologic eradication (defined as no growth of the causative pathogen from any samples taken [e.g., sputum, throat swab or bronchoalveolar lavage fluid] after treatment, regardless of the clinical outcome), change of CPIS from baseline after treatment (∆CPIS), length of stay in ICU, duration of MV and adverse events of bronchospasm and nephrotoxicity. Discrepancies were identified and resolved through discussion.

### Quality assessment

The two investigators also independently assessed the quality of RCTs using the risk of bias tool recommended by the Cochrane Handbook for Systematic Reviews of Interventions^21^. We also used Jadad score to assess the quality of included trials^22^. The quality of evidence resulting from the present meta-analysis was evaluated using the Grading of recommendations assessment, development, and evaluation (GRADE) approach^23^. Publication bias was evaluated by visually inspecting funnel plots and modified Galbraith tests.

### Statistical analysis

The results from all relevant studies were combined to estimate the pooled risk ratio (RR) and associated 95% confidence intervals (CI) for dichotomous outcomes. As to the continuous outcomes, mean differences (MD) and 95% CI were estimated as effective results. Some studies reported median as the measure of treatment effect, with accompanying interquartile range (IQR). Before data analysis, we estimated mean from median and standard deviations (SD) from IQR using the methods described in previous studies^24^. Heterogeneity was tested by using the *I*^2^ statistic. An *I*^2^ < 50% was considered to indicate insignificant heterogeneity and a fixed-effect model was used, whereas a random-effect model was used in cases of significant heterogeneity (*I*^2^ > 50%) using the Mantel–Haenszel method^25^. Testing the robustness of our outcomes and exploring the potential influence factors, we performed sensitivity analyses by omitting one study in each turn to investigate the influence of a single study on the overall pooled estimate of each predefined outcome. Also, subgroup analyses were performed concerning the primary outcome by pooling studies with the following: (1) types of nebulizers (Jet or ultrasonic or vibrating nebulizer); (2) dose of NA (≥ 800 mg/day or < 800 mg/day); (3) proportion of patients with drug-resistant bacteria (including multidrug-resistant (MDR), extensively drug-resistant (XDR) or pan drug-resistant (PDR) bacteria) (100% or < 100%); (4) study design (blinded or un-blinded), and estimated models (fixed-effect or random effect models). All analyses were performed using Review Manager, Version 5.3. The quality assessment of the evidence was evaluated by GRADE profiler software version 3.6 (GRADE Working Group, 2004–2007).

## Results

### Searching results

**Figure 1:**
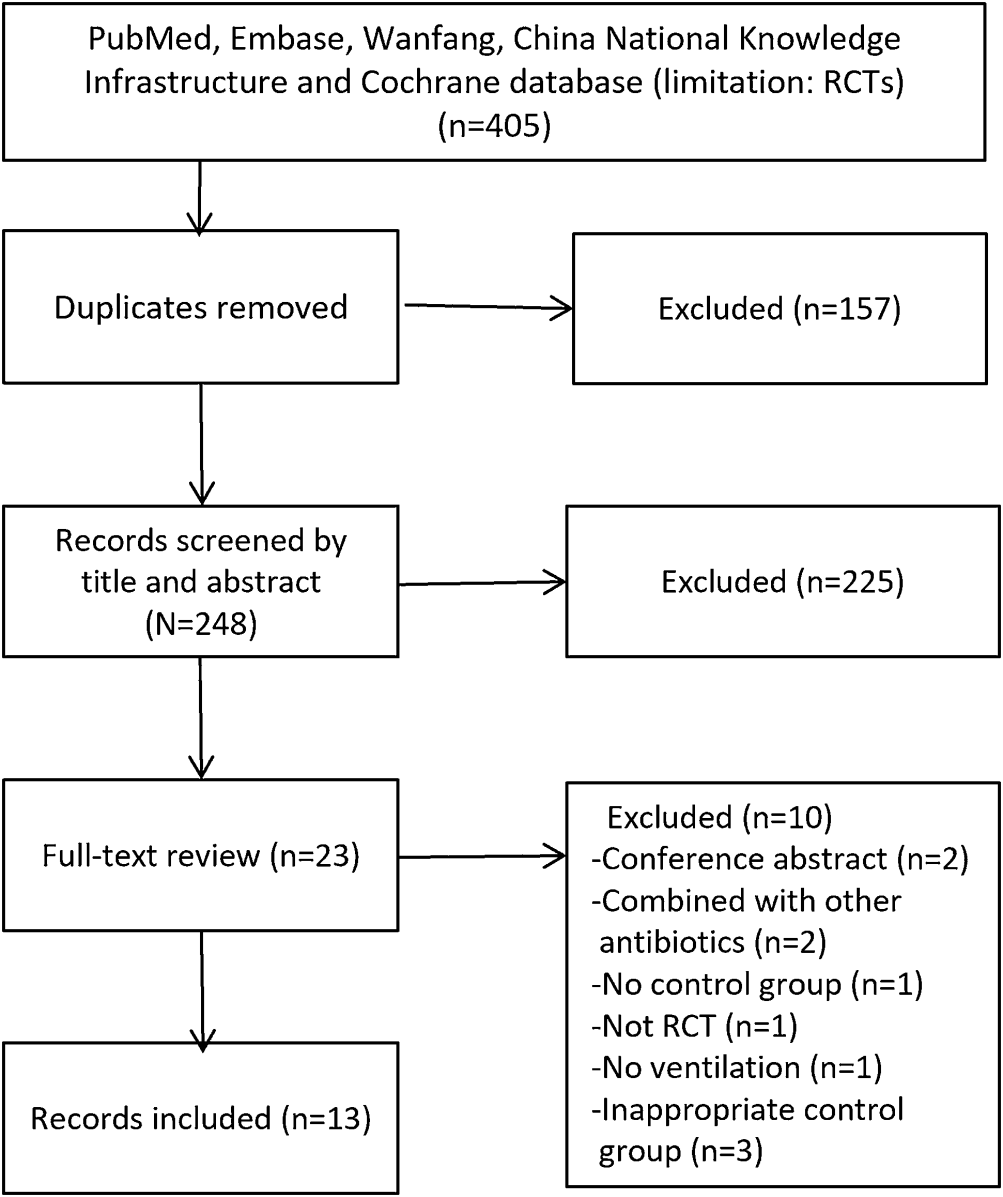
Selection process for RCTs included in the meta-analysis.

The literature search yielded 325 records through database searching, of which 24 full-text were considered for text-trials review. Finally, 13 RCTs with a total of 1733 patients met the inclusion criteria and were included in our study^11,14,18,19,26–34^ (Fig. 1). The details in the search strategy were shown in Appendix 2.

### Studies characteristics and quality assessment

The main characteristics of included RCTs and predefined outcomes are shown in Table 1 and Appendix 3, while the definitions of patient inclusion, microbiological cure criteria, and clinical response criteria are summarized in Appendix 4. All the included studies were conducted in medical-surgical ICUs. Ten^20,24,25,27–34^ out of the 13 RCTs were multicenter studies. A total of 1733 patients were included in intention-to-treat analysis while 1,450 patients were included in clinically evaluable. As to the type of nebulizer devices employed in the NA group, vibrating-mesh nebulizer (3 studies)^11,19,26^, ultrasonic nebulizer (2 study)^20,34^, and jet nebulizer (8 study)^14,24,25,27–33^ were used. During the treatment period, patients received concomitant intravenous antibiotics variable among the included studies, decided by the clinician, or based on pathogen-specific treatment criteria. Seven RCTs^11,14,19,20,26,28,33^ described in detail the nebulization technique, including nebulizer position, ventilator settings, humidifier, respiratory mode, and sedation during the nebulization period (Appendix 5).

**Table 1:** Characteristics of the studies included in current systemic review and meta-analysis.

Study	Study design	Type of pneumonia	Device for drug delivery	Patient characteristics (NA/Control)						NA regimen	Primary outcome
				No. of patients ITT^a^	No. of patients clinically evaluable	Age, mean, (year)	APACHE II mean	Patients with resistant GNP^b^ (%)	MV, IVAB or ICU/hospital stay before NA		
Niederman et al.^19^ , 2020	PR, DB, MC	HAP, HCAP, VAP, CAP	Vibrating mesh nebulizer	362/363	255/253	64/64	20/20	50/55	NR	400 mg every 12 h for 10 d	Survival at days 28–32
Ammar et al.^20^, 2018	PR, NB, SC	VAP	Ultrasound nebulizer	65/32	30/30	56/55	20/18	100/100	NR	20 mg/kg every 8 h	Clinical response
Chen^27^ 2018	PR, NB, SC	VAP	Jet nebulizer	55/55	55/55	73/73	13/13	NR	NR	400 mg every 12 h for 14 d	Clinical response and Bacteriological eradication
Liu et al.^14^, 2017	PR, DB, SC	VAP	Jet nebulizer	30/30	27/25	68/65	22/19	100/100	MV:17 vs. 18 d; ICU stay: 16 vs. 14 d	400 mg every 8 h for 7 d	Bacteriological eradication and new drug resistant to amikacin
Kollef et al.^11^, 2017	PR, DB, MC	VAP	Vibrating plate electronic nebulizer	71/72	71/71	58/62	19/19	45/29	IVAB: 7 vs. 5 d	300 mg twice daily for 10 d	Change from baseline in CPIS
Li et al.^28^, 2016	PR, NB, SC	VAP	Jet nebulizer	38/38	38/38	64/61	13/16	8/18	MV: 5 vs. 6 d; IVAB: 6 vs. 6 d	400 mg every 24 h for 7 d	Clinical response
Ji^29^ 2016	PR, NB, SC	VAP	Jet nebulizer	21/21	21/21	60/60	NR	100/100	NR	7.5 mg/kg every 12 h	Clinical response
Tong^30^ 2016	PR, NB, SC	VAP	Jet nebulizer	45/45	45/45	45/47	NR	NR	NR	600 mg every 24 h, for 7–14 d	Clinical response
Yue^31^ 2016	PR, NB, SC	VAP	Jet nebulizer	39/39	39/39	50/50	NR	NR	NR	600 mg every 24 h, for 14 d	Clinical response
Zhu^32^ et al., 2015	PR, NB, SC	VAP	Jet nebulizer	34/34	34/34	42/42	NR	NR	NR	7.5 mg/kg every 24 h for 8 d	Clinical response
Li et al.^33^, 2015	PR, NB, SC	VAP	Jet nebulizer	60/60	60/60	54/58	13/13	NR	MV: 5 vs. 6 d; IVAB: 31 vs. 22 d	400 mg every 12 h for 7 d	Clinical response
Niederman et al.^26^,2012	PR, DB, MC	HAP, VAP, CAP	Vibrating mesh nebulizer	47/22	47/22	59 /62	16/16	NR	ICU stay > 5 d: 94% vs. 82%; IVAB within two weeks: 85% vs. 86%	400 mg every 12 h or 24 h, for 7–14 d	Patients with Cmax ≥ 6,400 ug/mL and AUC0–24 h/256 ≥ 100
Meng^34^ 2011	PR, NB, SC	VAP	Ultrasound nebulizer	30/30	29/27	50/49	NR	100/100	NR	600 mg every 24 h for 10–14 d	Clinical response

APACHE II = acute physiology and chronic health evaluation II, AUC_0–24 h_ = area under the concentration–time curve from 0 to 24 h, CAP = community acquired pneumonia, CPIS = clinical pulmonary infection score, Cmax = maximum concentration, DB = double blind, GNB = gram‑negative pneumonia, HAP = hospital-acquired pneumonia, h = hours, HCAP = healthcare-associated pneumonia, ICU = intensive care unit, IVAB = intravenous antibiotics, MC = multi-centers, Mix-ICU = medical-surgical intensive care unit, NA = nebulized amikacin, NR = not reported, PR = prospective randomized, SD = standard deviation, SC = single-center, VAP = ventilator-associated pneumonia.

^a^ITT = intention-to-treat analysis, ^b^defined as multidrug‑resistant or extensively drug-resistant or pandrug-resistant gram‑negative pneumonia.

The Cochrane risk of bias score for each study is summarized in Appendix 6, Fig. S1A and S1b. Four studies^11,14,19,26^ were assessed to be at low risk of bias overall and nine studies^20,27–34^ were at high risk of bias overall. The median Jadad score of the included studies was 2.6 (range from 1 to 5, see Appendix 7). Using GRADE methodology, we evaluated the evidence for pooled data for clinical response rate, overall mortality, pneumonia associated mortality, microbiologic eradication, ∆CPIS, duration of MV, length of stay in ICU, nephrotoxicity, and bronchospasm to be moderate, moderate, moderate, low, low, very low, low, respectively (Table 2). Assessment of publication bias using visually inspecting funnel plots and modified Galbraith tests showed no potential publication bias among the included RCTs (Appendix 8, Fig. S2a and S2b) (Appendix 8, Fig. S2).

**Table 2:** Grading of recommendations assessment, development, and evaluation evidence profile for the role of adjunctive aerosolized amikacin in outcomes of the meta-analysis.

Outcome	No. of study	No. of patients	Relative effect (95% CI)	Estimated Absolute Effects	Heterogeneity *I*^2^ , (P)	Quality of the evidence (GRADE)*
Clinical response rate	13	1450	RR, 1.29 (1.14–1.47)	38 more per 1000 (from 10 fewer to 89 more)	49%, (0.02)	⊕⊕⊕○ Moderate because of risk of bias
Overall mortality	7	1058	RR, 1.17 (0.91, 1.50)	30 more per 1000 (from 16 fewer to 88 more)	0%, (0.77)	⊕⊕⊕○Moderate because of risk of bias
Pneumonia associated mortality	7	1066	RR, 1.12 (0.82, 1.52)	15 more per 1000 (from 23 fewer to 66 more)	0%, (0.87)	⊕⊕⊕○ Moderate because of risk of bias
Microbiologic eradication	11	921	RR, 1.51 (1.35, 1.69)	466 more per 1000 (from 163 fewer to 322 more)	6%, (0.38)	⊕⊕○○ Low because of risk of bias and inconsistency
Length of stay in ICU	4	785	–	Mean duration was 0.31 day lower (2.08 lower to 1.45 higher)	67%, (0.03)	⊕⊕○○ Low because of risk of bias and imprecision
∆Clinical pulmonary infection score	8	596	–	Mean difference was 1.08 lower (0.11 lower to 2.27 higher)	96%, (0.000)	⊕⊕○○ Low because of risk of bias and imprecision
Duration of mechanical ventilation	4	774	–	Mean duration was 0.45 day lower (2.69 lower to 1.78 higher)	84%, (0.0003)	⊕⊕⊕○ Very low because of risk of bias, inconsistency and imprecision
Nephrotoxicity	7	1026	RR, 0.82 (0.60, 1.12)	26 more per 1000 (from 57 fewer to 17 more)	2%, (0.41)	⊕⊕⊕○ Moderate because of risk of bias
Bronchospasm	8	1097	RR, 2.55 (1.40, 4.66)	38 more per 1000 (from 10 fewer to 89 more)	49%, (0.02)	⊕⊕⊕○ Moderate because of risk of bias

⊕⊕⊕○ moderate, ⊕⊕○○ low, ⊕○○○ very low, CI = confidence intervals, ICU = intensive care unit. RR = relative risk.

*GRADE Working Group grades of evidence.

High quality: Further research is very unlikely to change our confidence in the estimate of effect.

Moderate quality: Further research is likely to have an important impact on our confidence in the estimate of effect and may change the estimate.

Low quality: Further research is very likely to have an important impact on our confidence in the estimate of effect and is likely to change the estimate.

Very low quality: We are very uncertain about the estimate.

### Primary outcome

**Figure 2:**
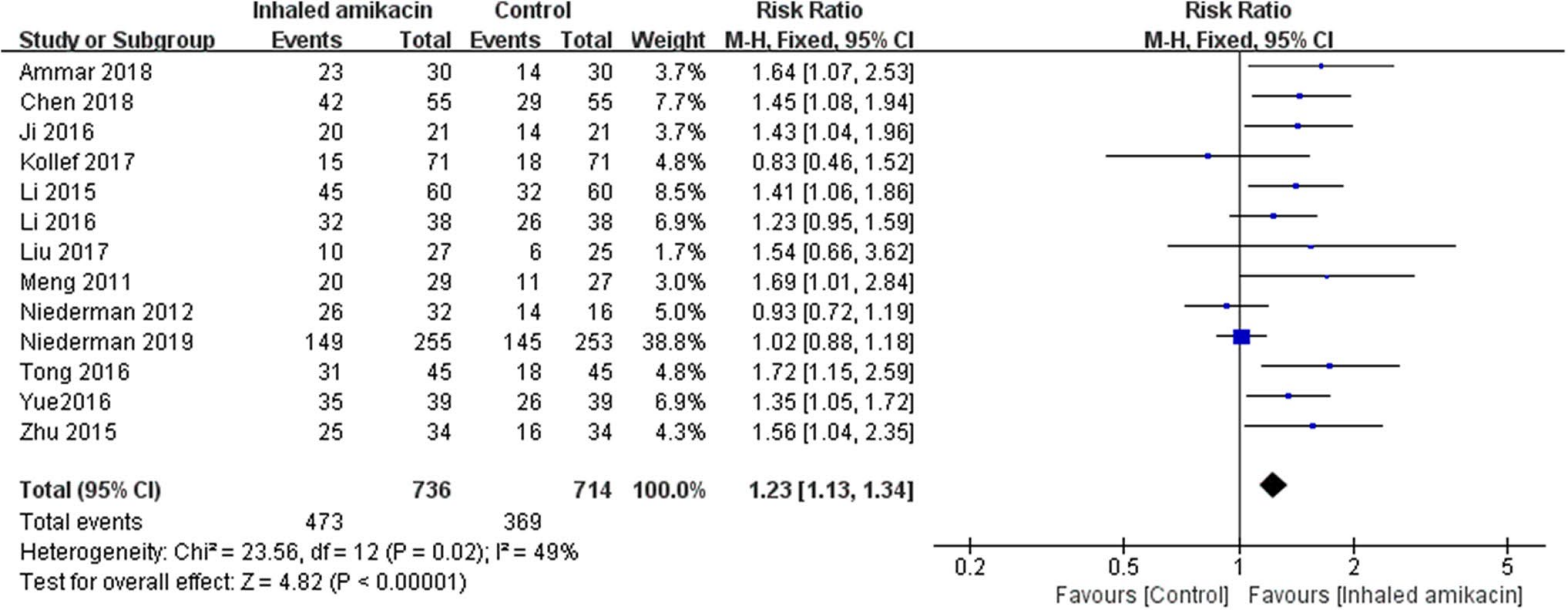
Forest plots of the effects of aerosolized amikacin on clinical response.

Clinical response rate was reported in all 13 RCTs^11,14,19,20,24–34^. The pooled analysis showed that, compared with control, NA improved clinical response (n = 1,450; RR = 1.24; 95% CI 1.13 to 1.35; *P* < 0.00001), with moderate heterogeneity (*I*^2^ = 47%) among the studies (Fig. 2). In the sensitivity analysis, exclusion of any single trial did not significantly alter the overall combined RR (*P* value ranging from 1.22 to 1.37, with *I*^2^ from 31 to 53%), whereas most subgroup analyses based on types of nebulizers, NA dose, sample size, study quality or study design confirmed similar improved clinical response among groups. However, the use of NA did not affect clinical response rate when pooling dada limited to studies using vibrating mesh nebulizer (*P* = 0.90), being high quality (*P* = 0.84), with large sample size (*P* = 0.15), or with blinding design (*P* = 0.84) (Table 3).

**Table 3:** Subgroup analysis on primary outcome of clinical response.

		Studies number	Patient number	Event in NA group	Event in control group	Risk ratio (95% CI)	*I*^2^	*P*
Types of nebulizers	Vibrating mesh nebulizer	3	698	191 of 358	177 of 340	0.99 [0.87, 1.13]	0%	0.90
	Ultrasonic nebulizer	2	114	23 of 59	14 of 57	1.64 [1.07, 2.53]	0%	0.003
	Jet nebulizer	8	778	255 of 390	185 of 388	1.37 [1.22, 1.55]	0%	< 0.0001
Patients with resistant GNP	100%	4	210	73 of 107	45 of 103	1.58 [1.24, 2.00]	0%	0.0002
	< 100%	9	1,240	401 of 629	324 of 611	1.24 [1.08, 1.42]	54%	0.003
Dose of NA	≥ 800 mg/day	7	652	301 of 462	254 of 460	1.25 [1.04, 1.51]	59%	0.02
	< 800 mg/day	7	542	173 of 272	129 of 270	1.33 [1.16, 1.53]	35%	< 0.0001
Sample size	< 100	9	570	223 of 295	145 of 275	1.28 [0.78, 2.12]	40%	< 0.0001
	> 100	4	880	251 of 441	224 of 439	1.19 [0.94, 1.50]	63%	0.15
Study quality	Low	9	700	273 of 351	186 of 349	1.46 [1.31, 1.63]	0%	< 0.0001
	High	4	750	201 of 385	183 of 365	1.01 [0.89, 1.16]	0%	0.84
Study design	Blinded	4	750	201 of 385	183 of 365	1.01 [0.89, 1.16]	0%	0.84
	Unblinded	9	700	23 of 30	14 of 30	1.46 [1.31, 1.63]	0%	< 0.0001

NA = nebulized amikacin; CI = confidence interval; GNP = gram‑negative pneumonia.

### Secondary outcome

There was no statistically significant differences between the NA and control groups in overall mortality (7 trials, n = 1,058; RR = 1.17; 95% CI 0.98 to 1.50; *I*^2^ = 0%; *P* = 0.21)^11,19,20,26,28,30,33^ (Appendix 9, Fig. S3) or pneumonia associated mortality (7 trials, n = 1,066; RR = 1.12; 95% CI 0.82 to 1.52; *I*^2^ = 0%; *P* = 0.48) [^11,14,19,26,28,30,33^ ,(Appendix 9, Fig. S4). The length of stay in ICU (4 trials, n = 785, MD = − 0.31 days; 95% CI − 2.08 to 1.45, *I*^2^ = 67%; *P* = 0.73)^11,20,26,28^ (Appendix 9, Fig. S5), duration of MV (4 studies, n = 774, MD = − 0.45 days; 95% CI − 2.69 to 1.78, *I*^2^ = 84%; *P* = 0.69)^11,19,20,28^ (Appendix 9, Fig. S6) and ∆CPIS (8 studies, n = 596, MD = 1.08; 95% CI − 0.11 to 2.27, *I*^2^ = 96%; *P* = 0.08)^11,14,20,29–32,34^ (Appendix 9, Fig. S7) were also similar. Eleven RCTs reported specific data on outcome of microbiologic eradication, with better microbiologic eradication using NA compared with control (11 studies, n = 921, RR = 1.32; 95% CI 1.09 to 1.59, *I*^2^ = 6%; *P* < 0.00001)^11,14,20,24–28,30–34^ (Appendix 9, Fig. S8). Further sensitivity analyses showed that exclusion of any single trial did not significantly alter the overall combined RR in all the secondary outcomes.

Eight studies presented data on bronchospasm during treatment, with 6.4% (36/562) and 2.4% (13/535) in NA and control groups^11,14,19,24^. Pooled the data showed significantly higher bronchospasm in the NA group (RR = 2.55; 95% CI 1.40–4.66; *I*^2^ = 0%; *P* = 0.002) (Appendix 9; Fig. S9). Nephrotoxicity was reported in eight studies^11,14,19,26,28–30,33^. In the study by Liu et al., the authors reported no significant difference in serum creatinine concentration between NA and placebo group at the time of randomization (*P* = 0.857) and day 7 (*P* = 0.614)^14^. The other seven studies reported renal failure rate, and pooled data showed no differences between the two groups (n = 1,026; RR = 0.82; 95% CI 0.60–1.12; *I*^2^ = 2%; *P* = 0.20)^11,19,26,28–30,33^ (Appendix 9, Fig. S10).

## Discussion

The present meta-analysis assessed the role of NA as adjunctive therapy in ventilated patients with GNP. We found NA has a better microbiologic eradication and improve the clinical response. Meanwhile, NA did not affect mortality, ∆CPIS, and duration of MV or ICU stay. Additionally, NA did not add significant nephrotoxicity, while it could cause more bronchospasm.

To date, several recent meta-analyses and guidelines have suggested favorable clinical response of aerosolized antibiotics in ventilated pneumonia^3–5^. However, pooled results of different study designs (RCTs and observational studies), various antibiotics (aminoglycosides, colistin, and vancomycin), and different therapy strategies (adjunctive and substitution) might contribute to the significant heterogeneity among the included studies. Meanwhile, observational studies have the risk of overrated pooled estimates. To address these limitations, we focused specifically on NA used as adjunctive therapy in ventilated GNP, expanded the sample size by including recent published RCTs, and conducted robust data analyses and quality evaluation. We found NA is effective as such a therapeutic strategy for GNP. Therefore, our findings support and expand the suggestions in previous meta-analyses and guidelines.

To facilitate comparison with the previous meta-analyses^4,5^, we chose clinical response as the primary outcome. Indeed, from a research and clinical standpoint, the clinical response may be a more reliable parameter compared with other important clinical outcomes (e.g., CPIS, microbiologic eradication or mortality, duration of MV, and ICU stay). For instance, the CPIS was originally designed for VAP diagnosis, rather than assessing the response to treatment^35^, whereas mortality is an outcome not only related to GNP, but it is also influenced by many other prognostic factors (e.g., underlying diseases, the severity of illness or immunity of the host). Furthermore, clinical response was the most reported outcome and might provide more evidence to aid in the clinical decision.

Our results showed NA exhibited better clinical response. However, we should interpret this finding with caution. First, we found moderate heterogeneity among the pooled trials in this outcome. This heterogeneity could be caused by different pathogenic bacteria and the definition of clinical response between the pooled trials. Subgroup-analysis of studies with large sample size and double blinding also could not confirm this benefit of NA. Second, we could not demonstrate a significant reduction in mortality, ICU LOS, and ventilated duration. Additionally, although NA resulted in better microbiologic eradication, the eradication data varied widely among the pooled studies (ranging from 29 to 71%)^11,14,18,24^, which means these data can be susceptible to some clinical factors, such as microbiological detection technique, the proportion of drug-resistant GNP, systemic antibiotics therapy, or airway secretions or antibiotics contained in bronchoalveolar lavage fluid. Of note, the positive detection of microbial culture may be affected by colonization with bacteria, and the correlation has been demonstrated to be poor between the positive cultures alone and histologically confirmed pneumonia^36^. Thus, microbiologic eradication based on microbial culture does not necessarily mean the eradication of deep parenchymal pneumonia.

Several included studies with high quality, though reporting the negative results, provided information concerning the specific treatments in NA. This might help to explain the opposite results among the included studies. On the one hand, the severity and extension of pulmonary infection might affect the lung deposition of NA. In ventilated animal models with pneumonia, lung tissue concentrations of NA were markedly lower in pulmonary segments with confluent pneumonia and lung abscess compared to that in the early stages of lung infection. However, most patients of included RCTs received NA only after their time-consuming VAP/GNP diagnosis procedures. This, to some extent, delays the administration of NA in the early stages of GNP. Furthermore, most of these patients also received a prolonged course of MV and/or intravenous amikacin before receiving NA. This might contribute to an increase in airway biofilms and bacterial resistance, thus making lung infection treatment more difficult and ineffective.

On the other hand, several critical factors, such as aerosol particle size, type of nebulizer, physical characteristics of the carrying gas, and respiratory settings during the implementation of NA can also influence lung deposition of NA. By and large, to increase the efficiency of aerosol delivery, ultrasonic or vibrating mesh nebulizers producing low flow turbulence, volume-control mode with the constant inspiratory flow and appropriate end-inspiratory pause (representing about 20% of the duty cycle) are preferred; whereas heating and humidification that increase the diameter of the aerosol particles (> 5 μm), decelerating flows, spontaneous modes or ventilator-patient asynchrony during NA period should be avoided. In one RCT focusing on nebulized antibiotics in VAP, the authors chose vibrating mesh nebulizers and filled out the well-designed checklist before NA to standardize and optimize the nebulization procedure. However, the total extrapulmonary (nebulizer chamber, the inspiratory limb of the respiratory circuit, and the expiratory filter) depositions of amikacin were as high as 40%. Therefore, it can be conceivable that in clinical practice, as shown in the included RCTs in the current study (Appendix 4), the efficiency of actual aerosol delivery may be lower. However, this may also mean that there is still ample space for improvement in nebulized techniques in the future.

This study has several limitations. First, most of included studies^14,18,24^ had a sample size of fewer than 100 patients, which might be subject to overestimation of effect size. Second, definitions and timing assessment of microbiologic eradication, the dose of amikacin used, as well as disease severity varied among the included RCTs. This might lead to observed heterogeneity, thus impairing the robustness of our findings. Third, the duration of MV before NA, time to start NA, and pathogens varied across included RCTs. The original plan of subgroup analysis to further explore trials based on the above diversities was hampered by insufficient data. Finally, the results of some subgroup analyses should be interpreted with caution due to insufficient studies, i.e., type of nebulizers or study design.

## Conclusion

In summary, based on the current evidence, the use of NA adjunctive to systemic antibiotics therapy showed better benefits in ventilated patients with GNP. However, the overall quality of included studies is poor and more well-designed RCTs are still needed to confirmed our results.

## Supplementary Information

Supplementary Information

## Abbreviations

CPIS: Clinical pulmonary infection score
CI: Confidence interval
GNP: Gram-negative pneumonia
GRADE: Grading of recommendations assessment, development, and evaluation
ICU: Intensive care unit
MD: Mean difference
MV: Mechanical ventilation
NA: Nebulized amikacin
RR: Risk ratio
RCTs: Randomized controlled trials
SD: Standard deviations
