# Supplementary material for: Amikacin nebulization for the adjunctive therapy of gram-negative pneumonia in mechanically ventilated patients: a systematic review and meta-analysis of randomized controlled trials
Source: Sci Rep. 2021 Mar 26;11:6969. doi: 10.1038/s41598-021-86342-8 (PMC7997905; doi:10.1038/s41598-021-86342-8)
Supplement: Supplementary file 1 — Supplementary Information [file 41598_2021_86342_MOESM1_ESM.docx]

**Amikacin nebulization for the adjunctive therapy of gram-negative pneumonia in mechanically ventilated patients: A systematic review and meta-analysis of randomized controlled trials**

**Jun-Ping Qin, MD; Hui-Bin Huang^*^, MD; Hua Zhou, MD, Yuan Zhu, MD; Yuan Xu, MD; Bin Du, MD**

***Corresponding author:**

**Hui-Bin Huang,** Department of Critical Care Medicine, Beijing Tsinghua Changgung Hospital, School of Clinical Medicine, Tsinghua University, Beijing 102218, China; Email: [hhba02922@btch.edu.cn](mailto:hhba02922@btch.edu.cn).

**Appendix files**

Appendix 1 PRISMA checklist…………………………………………………………………………………………………..…………………..2

Appendix 2 Search Strategy……………………………………………………………………………………………………………..…………..5

Appendix 3 Table S1 Summary of predefined outcomes of included studies………………….…………….………..……9

Appendix 4 Table S2 Definition of criteria for patient population, microbiological eradication and clinical

response…………………………………………………………………………………………………………………..……….…...10

Appendix 5 Table S3 Administration of nebulization technique in the included studies……………………..………12

Appendix 6 Fig S1 Risk of bias……………………………………………………………………………………………………….…..………14

Appendix 7 Table S4 The quality of the included studies assessed by the JADAD score……………………..………15

Appendix 8 Tests for publication bias for RR of the clinical response.………………………………………….……..………16

Appendix 9 Forest plots of the effects of Amikacin nebulization on the secondary outcomes……………………17

**Appendix 1**

**PRISMA 2009 checklist**

| **Section/topic** | **#** | **Checklist item** | **Reported on page #** |
| --- | --- | --- | --- |
| **TITLE** | | |  |
| Title | 1 | Identify the report as a systematic review, meta-analysis, or both. | 1 |
| **ABSTRACT** | | |  |
| Structured summary | 2 | Provide a structured summary including, as applicable: background; objectives; data sources; study eligibility criteria, participants, and interventions; study appraisal and synthesis methods; results; limitations; conclusions and implications of key findings; systematic review registration number. | 2 |
| **INTRODUCTION** | | |  |
| Rationale | 3 | Describe the rationale for the review in the context of what is already known. | 3-4 |
| Objectives | 4 | Provide an explicit statement of questions being addressed with reference to participants, interventions, comparisons, outcomes, and study design (PICOS). | 4 |
| **METHODS** | | |  |
| Protocol and registration | 5 | Indicate if a review protocol exists, if and where it can be accessed (e.g., Web address), and, if available, provide registration information including registration number. | 5 |
| Eligibility criteria | 6 | Specify study characteristics (e.g., PICOS, length of follow-up) and report characteristics (e.g., years considered, language, publication status) used as criteria for eligibility, giving rationale. | 6 |
| Information sources | 7 | Describe all information sources (e.g., databases with dates of coverage, contact with study authors to identify additional studies) in the search and date last searched. | 6 |
| Search | 8 | Present full electronic search strategy for at least one database, including any limits used, such that it could be repeated. | 6 and Appendix 2 |
| Study selection | 9 | State the process for selecting studies (i.e., screening, eligibility, included in systematic review, and, if applicable, included in the meta-analysis). | 6 |
| Data collection process | 10 | Describe method of data extraction from reports (e.g., piloted forms, independently, in duplicate) and any processes for obtaining and confirming data from investigators. | 7 |
| Data items | 11 | List and define all variables for which data were sought (e.g., PICOS, funding sources) and any assumptions and simplifications made. | 7 |
| Risk of bias in individual studies | 12 | Describe methods used for assessing risk of bias of individual studies (including specification of whether this was done at the study or outcome level), and how this information is to be used in any data synthesis. | 7-8 |
| Summary measures | 13 | State the principal summary measures (e.g., risk ratio, difference in means). | 8 |
| Synthesis of results | 14 | Describe the methods of handling data and combining results of studies, if done, including measures of consistency (e.g., I^2^) for each meta-analysis. | 8 |

| Risk of bias across studies | 15 | Specify any assessment of risk of bias that may affect the cumulative evidence (e.g., publication bias, selective reporting within studies). | 8-9 |
| --- | --- | --- | --- |
| Additional analyses | 16 | Describe methods of additional analyses (e.g., sensitivity or subgroup analyses, meta-regression), if done, indicating which were pre-specified. | 8-9 |
| **RESULTS** | | |  |
| Study selection | 17 | Give numbers of studies screened, assessed for eligibility, and included in the review, with reasons for exclusions at each stage, ideally with a flow diagram. | 10 |
| Study characteristics | 18 | For each study, present characteristics for which data were extracted (e.g., study size, PICOS, follow-up period) and provide the citations. | 10 |
| Risk of bias within studies | 19 | Present data on risk of bias of each study and, if available, any outcome level assessment (see item 12). | 10-11 |
| Results of individual studies | 20 | For all outcomes considered (benefits or harms), present, for each study: (a) simple summary data for each intervention group (b) effect estimates and confidence intervals, ideally with a forest plot. | 11-12 |
| Synthesis of results | 21 | Present results of each meta-analysis done, including confidence intervals and measures of consistency. | 11-12 |
| Risk of bias across studies | 22 | Present results of any assessment of risk of bias across studies (see Item 15). | 10-11 |
| Additional analysis | 23 | Give results of additional analyses, if done (e.g., sensitivity or subgroup analyses, meta-regression [see Item 16]). | 11-12 |
| **DISCUSSION** | | |  |
| Summary of evidence | 24 | Summarize the main findings including the strength of evidence for each main outcome; consider their relevance to key groups (e.g., healthcare providers, users, and policy makers). | 13 |
| Limitations | 25 | Discuss limitations at study and outcome level (e.g., risk of bias), and at review-level (e.g., incomplete retrieval of identified research, reporting bias). | 17 |
| Conclusions | 26 | Provide a general interpretation of the results in the context of other evidence, and implications for future research. | 18 |
| **FUNDING** | | |  |
| Funding | 27 | Describe sources of funding for the systematic review and other support (e.g., supply of data); role of funders for the systematic review. | 24 |

**Appendix 2**

**Search Strategy** (Limited to RCT

Database: PubMed Embase Cochrane library

Search completed 20th Jun 2021

----------------------------------------------------------------------------------------------------------------------

**PubMed**

#1“Aerosols” [Mesh]

#2 Administration, Inhalation [mesh]

#3“Nebulizers and Vaporizers” [mesh]

#4 nebul*[tiab]

#5 aerosol*[tiab]

#6 vaporiz*[tiab]

#7 inhal*[tiab]

#8 pulmonary delivery*[tiab]

#9 atomiz*[tiab]

#10 #1 OR #2 OR #3 OR #4 OR #5 OR #6 OR #7 OR #8 #9

[Result: 181,103]

#11 controlled clinical trial* [pt]

#12 randomized controlled trial [pt]

#13 random* [tiab]

#14 placebo* [tiab]

#15 clinical trials as topic [mesh: noexp]

#16 trial [tiab]

#17 animals [mh] NOT humans [mh]

#18 #11 OR #12 OR #13 OR #14 OR #15 OR #16 NOT #17

[Result: 1322,509]

#19 “Pneumonia, Ventilator-Associated” [mesh]

#20 “healthcare-associated pneumonia”[mesh]

#21 ventilator associated pneumonia*[tiab]

#22 VAP[tiab]

#23 nosocomial pneumonia*[tiab]

#24 Hospital-acquired pneumonia*[tiab]

#25 HAP[tiab]

#26 viral respiratory infection*[tiab]

#27 fungal respiratory infection*[tiab]

#28 ventilat*[tiab]

#29 intubat*[tiab]

#30 lung infect*[tiab]

#31 respiratory tract*[tiab]

#32 #19 OR #20 OR #21 OR #22 OR #23 OR #24 OR #25 OR #26 OR #27 OR #28 OR #29 OR #30 #

[Result: 1327,518]

#33 amikacin[mesh]

#34 aminoglycosides[mesh]

#35 amikacin*[tiab]

#36 aminoglycoside*[tiab]

#37 #33 OR #34 OR #35 OR #36

[Result: 172,061]

#38 #10 AND #18 AND #32 AND #37

[Result: 61]

----------------------------------------------------------------------------------------------------------------------

**Embase**

No. Query, Results

#33 #11 AND #12 AND #27 AND #32 [Result: 144]

#32 #28 OR #29 OR #30 OR #31 69972

#31 'aminoglycoside*':ab,ti AND [embase]/lim

#30 'amikacin*':ab,ti AND [embase]/lim

#29 'aminoglycoside'/exp

#28 'amikacin'/exp

#27 #13 OR #14 OR #15 OR #16 OR #17 OR #18 OR #19 OR #20 OR #21 OR #22 OR #23 OR #24 OR #25 OR #26 [Result: 386915]

#26 'respiratory tract*':ab,ti AND [embase]/lim

#25 'lung infect*':ab,ti AND [embase]/lim

#24 'intubat*':ab,ti AND [embase]/lim

#23 'ventilat*':ab,ti AND [embase]/lim

#22 'fungal respiratory infection*':ab,ti AND [embase]/lim

#21 'viral respiratory infection*':ab,ti AND [embase]/lim

#20 'cap':ab,ti AND [embase]/lim

#19 'hap':ab,ti AND [embase]/lim

#18 'hospital-acquired pneumonia*':ab,ti AND [embase]/lim

#17 'nosocomial pneumonia*':ab,ti AND [embase]/lim

#16 'vap':ab,ti AND [embase]/lim

#15 'ventilator associated pneumonia*':ab,ti AND [embase]/lim

#14 'health care associated pneumonia'/exp

#13 'ventilator associated pneumonia'/exp

#12 'clinical trial'/exp OR 'randomization'/exp OR 'single blind procedure'/exp OR 'double blind procedure'/exp OR 'randomized controlled trial'/exp OR 'crossover procedure'/exp OR 'placebo'/exp OR 'prospective studies'/exp OR ('randomi?ed controlled' NEXT/1 trial*) OR rct OR 'randomly allocated' OR 'allocated randomly' OR 'random allocation' OR (allocated NEAR/2 random) OR (single NEXT/1 blind*) OR (double NEXT/1 blind*) OR ((treble OR triple) NEAR/1 blind*) OR placebo* [Result: 2476243]

#11 #1 OR #2 OR #3 OR #4 OR #5 OR #6 OR #7 OR #8 OR #9 OR #10 [Result: 242957]

#10 'atomiz*':ab,ti AND [embase]/lim

#9 'pulmonary delivery*':ab,ti AND [embase]/lim

#8 'inhal*':ab,ti AND [embase]/lim

#7 'vaporiz*':ab,ti AND [embase]/lim

#6 'aerosol*':ab,ti AND [embase]/lim

#5 'nebul*':ab,ti AND [embase]/lim

#4 'nebulizer'/exp

#3 'inhalational drug administration'/exp

#2 'aerosol'/exp

#1 aerosols:ab,ti AND [embase]/lim

----------------------------------------------------------------------------------------------------------------------

**Cochrane library**

ID Search

#1 MeSH descriptor: [Aerosols] explode all trees

#2 MeSH descriptor: [Inhalation] explode all trees

#3 MeSH descriptor: [Nebulizers and Vaporizers] explode all trees

#4 (nebul*):ti,ab,kw (Word variations have been searched)

#5 (aerosol*):ti,ab,kw (Word variations have been searched)

#6 (vaporiz*):ti,ab,kw (Word variations have been searched)

#7 (inhal*):ti,ab,kw (Word variations have been searched)

#8 (pulmonary delivery*):ti,ab,kw (Word variations have been searched)

#9 (atomiz*):ti,ab,kw (Word variations have been searched)

#10 #1 OR #2 OR #3 OR #4 OR #5 OR #6 OR #7 OR #8 OR #9

#11 MeSH descriptor: [Pneumonia, Ventilator-Associated] explode all trees

#12 MeSH descriptor: [Healthcare-Associated Pneumonia] explode all trees

#13 (ventilator associated pneumonia*):ti,ab,kw (Word variations have been searched)

#14 (VAP):ti,ab,kw (Word variations have been searched)

#15 (nosocomial pneumonia*):ti,ab,kw (Word variations have been searched)

#16 (Hospital-acquired pneumonia*):ti,ab,kw (Word variations have been searched)

#17 (CAP):ti,ab,kw (Word variations have been searched)

#18 (viral respiratory infection*):ti,ab,kw (Word variations have been searched)

#19 (fungal respiratory infection*):ti,ab,kw (Word variations have been searched)

#20 (ventilat*):ti,ab,kw (Word variations have been searched)

#21 (intubat*):ti,ab,kw (Word variations have been searched)

#22 (lung infect*):ti,ab,kw (Word variations have been searched)

#23 (respiratory tract*):ti,ab,kw (Word variations have been searched)

#24 (HAP):ti,ab,kw (Word variations have been searched)

#25 #11 OR #12 OR #13 OR #14 OR #15 OR #16 OR #17 OR #18 OR #19 OR #20 OR #21 OR #22 OR #23 OR #24

#26 MeSH descriptor: [Amikacin] explode all trees

#27 (amikacin*):ti,ab,kw (Word variations have been searched)

#28 (aminoglycoside*):ti,ab,kw (Word variations have been searched)

#29 MeSH descriptor: [Aminoglycosides] explode all trees

#30 #26 OR #27 OR #28 OR #29

#31 #10 AND #25 AND #30

[Results: 170]

------------------------------------------------------------------------------------------------------------------------

Wanfang database

主题：（危重症 or 重症监护or 重症 or ICU or 呼吸机 or 机械通气）and 主题：（阿米卡星雾化）and 主题：（随机对照 or 随机 or RCT）

[Results: 13]

------------------------------------------------------------------------------------------------------------------------

China National Knowledge Infrastructure database

TKA=“阿米卡星雾化” and TKA=("危重症 + “重症监护” + “重症” + “ICU” + “呼吸机” + “机械通气”）and TKA=("随机对照" + "随机" + "RCT"）

[Results: 17]

------------------------------------------------------------------------------------------------------------------------

**Studies needed for full-reviewed but not included in the current meta-analysis (n=10 trials)**

1. Lu Q, Yang J, Liu Z, et al. Nebulized ceftazidime and amikacin in ventilator-associated pneumonia caused by Pseudomonas aeruginosa. American journal of respiratory and critical care medicine. 2011;184(1):106-115.

2. A Bruce Montgomery, Shirley Vallance, Tammy Abuan, et al. A Randomized Double-Blind Placebo-Controlled Dose-Escalation Phase 1 Study of Aerosolized Amikacin and Fosfomycin Delivered via the PARI Investigational eFlow® Inline Nebulizer System in Mechanically Ventilated Patients. J Aerosol Med Pulm Drug Deliv . 2014 Dec;27(6):441-8.

3. Hassan NA, Awdallah FF, Abbassi MM, et al. Nebulized Versus IV Amikacin as Adjunctive Antibiotic for Hospital and Ventilator-Acquired Pneumonia Postcardiac Surgeries: A Randomized Controlled Trial. Critical care medicine. 2018;46(1):45-52.

4. Brian H Gibson, John P Sharpe, Richard H Lewis, et al. Use of Aerosolized Antibiotics in Gram-Negative Ventilator-Associated Pneumonia in Trauma Patients. Am Surg . 2018 Dec 1;84(12):1906-1912.

5. Yaroshetskiy, A. Rezepov, N. Mandel, I. Khodak, V. Konanykhin, V. Influence of amikacin inhalation on the efficacy of ventilation-associated pneumonia and ventilation-associated tracheobronchitis treatment caused by multi-drug resistant gram-negative bacteria: Comparative study. Critical Care. 2018 22. Conference Abstract.

6. DE Griffith, G Eagle, R Thomson, et al. Amikacin Liposome Inhalation Suspension for Treatment-Refractory Lung Disease Caused by Mycobacterium avium Complex (CONVERT). A Prospective, Open-Label, Randomized Study. American journal of respiratory and critical care medicine, 2018, 198(12), 1559‐1569.

7. Schreiber, A. F. Arana, H. Lu, et al. Nebulized versus intravenous amikacin in ventilator-associated pneumonia Caused by pseudomonas aeruginosa. European Respiratory Journal. 2016 48. Conference Abstract.

8. Palmer LB, Smaldone GC. Reduction of bacterial resistance with inhaled antibiotics in the intensive care unit. American journal of respiratory and critical care medicine. 2014;189(10):1225-1233.

9. Chen qing，Chen li-juan，Liu yue-jian. Clinical analysis on the efficacy of amikacin atomization inhalation combined with intrave- nous anti-infection treatment for hospital-acquired pneumonia with multiple drug-resistant bacteria infection. Shi Yong Yi yuan Lin Chuang Za Zhi. 2016, 7(13), 114-117.

10. Clinical effect of amikacin atomization inhalation in the treatment of ventilation-associated pneumonia caused by pseudomonas aeruginosa. China Medical Engineering, 2012, 20(4), 156-157.

11. Yang chun-hui, Li Yong, Xie Yang-yong et al. Characteristics of airway secretion and blood pharmacokinetics of amikacin inhalation. Nei Ke Ji Wei Zhong Zheng Za Zhi. 2013, 19(3), 165-167.

**Appendix 3**

**Table S1 Summary of predefined outcomes of included studies**

| Study / Year | Clinical response (%) | | Overall mortality (%) | | Pneumonia associated mortality (%) | | Microbiologic eradication (%) | | Length of stay in ICU  mean (SD), (day) | | Duration of mechanical ventilation  mean (SD), (day) | | Change in clinical pulmonary  infection score | |
| --- | --- | --- | --- | --- | --- | --- | --- | --- | --- | --- | --- | --- | --- | --- |
|  | AA | Ctrl | AA | Ctrl | AA | Ctrl | AA | Ctrl | AA | Ctrl | AA | Ctrl | AA | Ctrl |
| Niederman et al^19^ , 2020 | 58.4 | 57.3 | 25.1 | 22.5 | 16.9 | 14.2 | NA | NA | 21.3 (8.2) | 21.9 (8.0) | 20.6 (10.1) | 20.2 (10.2) | NA | NA |
| Ammar et al^20^, 2018 | 76.7 | 46.7 | NA | NA | 16.7 | 26.7 | 30 | 33.3 | 5.32 (1.9) | 7.3 (2.1) | 11.9 (2.6) | 15.3 (3.1) | 4.5 (0.7) | 3 (0.7) |
| Chen^27^ 2018 | 76.4 | 52.7 | NA | NA | NA | NA | 74.5 | 41.5 | NA | NA | NA | NA | NA | NA |
| Liu et al^14^, 2017 | 37 | 24 | 22.2 | 32 | NA | NA | 40.7 | 16 | NA | NA | NA | NA | 4.2 (1.6) | 5.8 (2.1) |
| Kollef et al^11^, 2017 | 21.1 | 25.4 | 23.9 | 16.9 | 14.1 | 14.1 | 83.1 | 59.2 | 28.9 (12.4) | 26.2 (15.6) | 18.2 (9.7) | 15.5 (9.7) | 0.6 (2.7) | 0.7 (3) |
| Li et al^28^, 2016 | 84.2 | 68.4 | 15.8 | 10.3 | NA | NA | 711 | 39.5 | 12 (44.4) | 12 (5.93) | 9 (4.44) | 8.5 (3) | NA | NA |
| Ji^29^ 2016 | 95.2 | 66.7 | NA | NA | NA | NA | NA | NA | NA | NA | NA | NA | 2.8 (2.4) | 1.3 (2.6) |
| Tong^30^ 2016 | 68.9 | 40 | 6.7 | 8.9 | NA | NA | 73.3 | 53.3 | NA | NA | NA | NA | 5.7 (1.3) | 4.5 (1.9) |
| Yue^31^ 2016 | 89.7 | 66.7 | NA | NA | NA | NA | 76.9 | 41 | NA | NA | NA | NA | 7.7 (1.1) | 3.5 (1.1) |
| Zhu^32^ et al, 2015 | 73.5 | 47.1 | NA | NA | NA | NA | 82.3 | 55.9 | NA | NA | NA | NA | 5.8 (1.4) | 5.2 (1.3) |
| Li et al^33^, 2015 | 75 | 53.3 | 10 | 6.7 | NA | NA | 75 | 43.3 | NA | NA | NA | NA | NA | NA |
| Niederman et al^26^,2012 | 84.4 | 87.5 | 21.2 | 9.1 | 4.2 | 0 | 68.1 | 63.6 | NA | NA | NA | NA | NA | NA |
| Meng^34^ 2011 | 69 | 40.7 | NA | NA | NA | NA | 72.4 | 55.6 | NA | NA | NA | NA | 5.7 (1.3) | 4.5 (1.8) |

AA = aerosolized amikacin group, Ctrl = control group, ICU = intensive care unit, NA = not available, SD = standard deviation

**Appendix 4**

**Table S2 Definition of criteria for patient population, microbiological eradication and clinical response**

| Study / Year | Patient population | Microbiologic eradication | Clinical response |
| --- | --- | --- | --- |
| Niederman et al^19^ , 2020 | Pneumonia caused by Gram-negative pathogens in intubated and mechanically ventilated patients | The absence of the original pathogen(s) at the post-treatment TOC or LFU culture of specimens from the original site of infection (tracheal aspirate/sputum) | Early clinical response was a composite endpoint based on CPIS on days 3, 5, and 10 (vs. baseline CPIS); the presence of empyema or lung abscess at days 3, 5, or 10; and all-cause mortality up to the EOT visit. Early clinical response was considered to have been attained if success criteria for all three CPIS determinations were met, and if patients had not died or developed empyema or lung abscesses |
| Ammar et al^20^, 2018 | Patients having ventilator-associated pneumonia (VAP) with positive sputum culture showing Gram-negative MDRO | No growth in the final culture during hospitalization | Clinical cure (i.e., resolution of infection symptoms and signs by the end of antibiotic treatment) and clinical improvement (i.e., partial resolution of infection symptoms and signs) |
| Chen^27^ 2018 | Patients with mechanically-ventilated and pneumonia caused by Gram-negative bacteria | Not report | Not report |
| Liu et al^14^, 2017 | Ventilated patients with confirmed MDR‑GNB ventilator-associated pneumonia | Defined as no growth in culture and no visible organisms seen on Gram staining identified at randomization | Defined as the resolution of clinical and biological signs of infection |
| Kollef et al^11^, 2017 | Eligible patients were intubated and mechanically-ventilated with a diagnosis of pneumonia caused by Gram-negative bacteria | Tracheal culture at Day 7 negative for Gram-negative bacteria | Defined as both absence of Gram-negative bacteria [negative culture or no sputum available to culture in an extubated patient at Day 14 or earlier] and CPIS of <6 at Day 14 or earlier |
| Li et al^28^, 2016 | Patients with ventilation-associated pneumonia caused by pseudomonas aeruginosa | Tracheal culture at Day 7 negative for pseudomonas aeruginosa | Defined as the improvement or recover to normal of the clinical symptoms and biochemical indicators of lung infection. |
| Ji^29^ 2016 | Patients with ventilation-associated pneumonia caused by MDR-pseudomonas aeruginosa | Not report | Defined as (1) marked effect: the complete resolution of clinical symptoms and sign, and the complete absorption of lesions. (2) effective: the significant resolution of clinical symptoms and sign, and the significant absorption of lesions. Total effective rate = (marked effect + effective) / total number of cases × 100% |
| Tong^30^ 2016 | Patients with mechanically-ventilated and pneumonia caused by Gram-negative bacteria | Not report | Defined as body temperature and white blood cells returned to normal, no purulent secretions, and shadows of lung infiltration disappeared |
| Yue^31^ 2016 | Patients with mechanically-ventilated and pneumonia caused by Gram-negative bacteria | Defined as (1) removal: No growth in the final culture; (2) assumed removal: the secretions are too small to be easily removed, and resolution of symptoms, signs, and lab tests. Total Microbiologic eradication = removal + assumed removal | Defined as (1) marked effect: the complete resolution of clinical symptoms and sign, and the complete absorption of lesions. (2) effective: the significant resolution of clinical symptoms and sign, and the significant absorption of lesions. Total effective = marked effect + effective |
| Zhu^32^ et al, 2015 | Patients with ventilation-associated pneumonia caused by acinetobacter baumannii | No growth in the final culture in Bronchoalveolar lavage fluid during hospitalization | Defined as body temperature and white blood cells returned to normal, no purulent secretions, and shadows of lung infiltration disappeared |
| Li et al^33^, 2015 | Patients with mechanically-ventilated and pneumonia caused by Gram-negative bacteria | The absence of pathogen after treatment | Defined as the improvement or recover to normal of the clinical symptoms and biochemical indicators of lung infection. |
| Niederman et al^26^,2012 | Adults expected to be on mechanical ventilation for ≥3 days were included if they had a clinical diagnosis of Gram-negative hospital-acquired pneumonia, VAP, or HCAP; had a clinical pulmonary infection score (CPIS) of ≥6 | Confirmed eradication of the original pathogen or presumed eradication in patients with complete or partial resolution of pneumonia | Complete or partial resolution of signs and symptoms of pneumonia, improvement or lack of progression of all abnormalities on chest X-ray, and no additional intravenous antibiotics since completion of study treatment |
| Meng^34^ 2011 | Ventilated patients with confirmed MDR‑GNB ventilator-associated pneumonia | Defined as (1) removal: No growth in the final culture; (2) assumed removal: the secretions are too small to be easily removed, and resolution of symptoms, signs, and lab tests. Total Microbiologic eradication = removal + assumed removal | Defined as complete resolution of signs and symptoms of pneumonia, improvement of all abnormalities on chest X-ray, and body temperature and white blood cells returned to normal |

**Appendix 5**

**Table S3 Administration of nebulization technique in the included studies**

| Study | Nebulization technique |
| --- | --- |
| Niederman et al^19^ , 2020 | The nebulizer is positioned close to the endotracheal tube, minimizing the area of tubing on which aerosolized drug may be lost. The system has achieved an estimated lung dose of ~50% of the nominal dose in the nebulizer (based on drug collected at the lower end of the endotracheal tube), in bench models of mechanical ventilation. The dose of amikacin delivered is also unaffected by circuit humidification. During mechanical ventilation, synchronization of nebulization delivers the specially formulated amikacin solution only during the first 75% of the patient’s inspiration, enabling a high dose to be delivered into the lung while limiting drug losses during exhalation. The system can be used with standard, adult ventilator settings and does not require patient sedation, or changes to the inspiratory-to-expiratory ratio or other ventilator settings, unlike some generic systems. |
| Ammar et al^20^, 2018 | Nebulization was performed with an ultrasound nebulizer positioned on the inspiratory limb proximal to the Y-piece. To reduce flow turbulences and extrapulmonary deposition, specific ventilator settings were used during the nebulization period. They included removal of the humidifier (heat and moisture exchanger), volume-controlled mode, constant inspiratory flow, respiratory rate of 12 breaths/min, inspiratory: expiratory ratio of 1-2, tidal volume of 8 ml/kg, and an end-inspiratory pause representing 20% of the duty cycle. Synchrony between the patient and the ventilator was guaranteed by deepening the level of sedation to avoid inspiratory turbulences and optimize distal lung deposition of nebulized particles. |
| Chen^27^ 2018 | Not report |
| Liu et al^14^, 2017 | Amikacin or placebo was nebulized through a jet nebulizer (BD Medical Technology, USA). The following ventilator settings were followed during nebulization: volume control mode, tidal volume of 8 ml/kg, constant inspiratory flow rate of 40 L/min, nebulization set during inspiration, with heat and moisture exchangers remove or heated humidifiers off, inspiratory to expiratory (I:E) ratio ≤ 50%, and an end‑inspiratory pause of 20% of the duty cycle. To optimize the synchronization of the patients and the nebulizer, the patients were sedated with propofol and fentanyl during treatment. |
| Kollef et al^11^, 2017 | AFIS/placebo were administered with an investigational inline vibrating plate electronic nebulizer (PARI GMBH, Starnburg, Germany) placed proximal to the ventilator Y-connector and run continuously over approximately 12 minutes. Humidity was maintained during treatment. Any ventilator model was allowed as long as the bias flow was <4 L/min. Ventilator settings were not changed and the nebulizer was left in place during the AFIS/placebo treatment period. |
| Li et al^28^, 2016 | Nebulization was performed using the function of according ventilator. The humidification function of the ventilator was turned off during nebulization. Nebulization lasted 30-60 minutes, for 7 days. If the patient weaning within 7 days, high-flow oxygen for nebulization was given. |
| Ji^29^ 2016 | Not report |
| Tong^30^ 2016 | Not report |
| Yue^31^ 2016 | Not report |
| Zhu^32^ et al, 2015 | Not report |
| Li et al^33^, 2015 | Nebulization was performed using the function of according ventilator. The humidification function of the ventilator was turned off during nebulization. Nebulization lasted 30-60 minutes, for 7 days. If the patient weaning within 7 days, high-flow oxygen for nebulization was given. |
| Niederman et al^26^,2012 | Tracheal aspirate concentrations of amikacin did not vary by ventilator mode, with 71% receiving assist-control ventilation and the remainder receiving pressure-support mode. |
| Meng^34^ 2011 | Nebulization was performed using the function of according ventilator. The following ventilator settings were followed during nebulization: volume control mode, Vt of 8 ml/kg, respiratory rate of 15/min, nebulization set during inspiration, with heat and moisture exchangers remove or heated humidifiers off, and an end‑inspiratory pause of 20% of the duty cycle. |

**Appendix 6**

**Fig S1a Risk of bias summary**


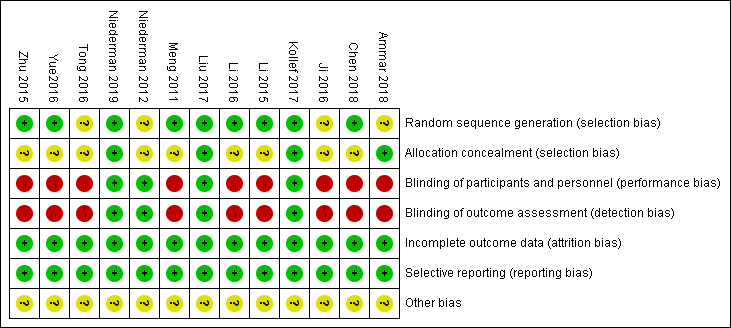


**Fig S1b Risk of bias graph**


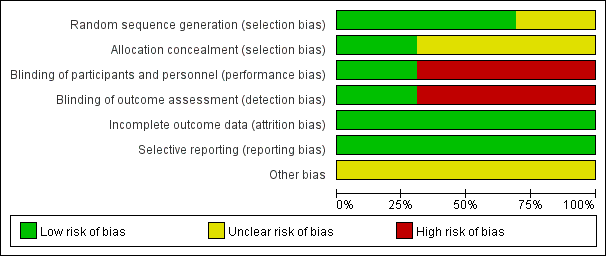


**Appendix 7**

**Table S4. The quality of the included studies assessed by the Jadad score**

| Studies | Was the study described as randomized? | Was the study described as double blind? | Was there a description of withdrawals and dropouts? | JADAD score* |
| --- | --- | --- | --- | --- |
| Niederman et al^19^ , 2020 | 2 | 2 | 1 | 5 |
| Ammar et al^20^, 2018 | 1 | 0 | 1 | 2 |
| Chen^27^ 2018 | 2 | 0 | 0 | 2 |
| Liu et al^14^, 2017 | 2 | 2 | 1 | 5 |
| Kollef et al^11^, 2017 | 2 | 2 | 1 | 5 |
| Li et al^28^, 2016 | 2 | 0 | 1 | 3 |
| Ji^29^ 2016 | 1 | 0 | 0 | 1 |
| Tong^30^ 2016 | 1 | 0 | 0 | 1 |
| Yue^31^ 2016 | 2 | 0 | 0 | 2 |
| Zhu^32^ et al, 2015 | 2 | 0 | 0 | 2 |
| Li et al^33^, 2015 | 2 | 0 | 1 | 3 |
| Niederman et al^26^,2012 | 1 | 2 | 1 | 4 |
| Meng^34^ 2011 | 2 | 0 | 1 | 3 |

*Scoring the items:

Ether give a score of 1 point for each "yes" or 0 points for each "no." There are no in-between marks.

Give 1 additional point if:
For question 1, the method to generate the sequence of randomization was described and it was appropriate (table of random numbers, computer generated). and / or: If for question 2, the method of double blinding was described and it was appropriate (identical placebo, active placebo, dummy, etc.).

Deduct 1 point if:
For question 1, the method to generate the sequence of randomization was described and it was inappropriate (patients were allocated alternately, or according to date of birth, hospital number, etc.). and / or: If for question 2, the study was described as double blind but the method of blinding was inappropriate (e.g., comparison of tablet vs. injection with no double dummy).

**Appendix 8. Tests for publication bias for RR of the clinical response**


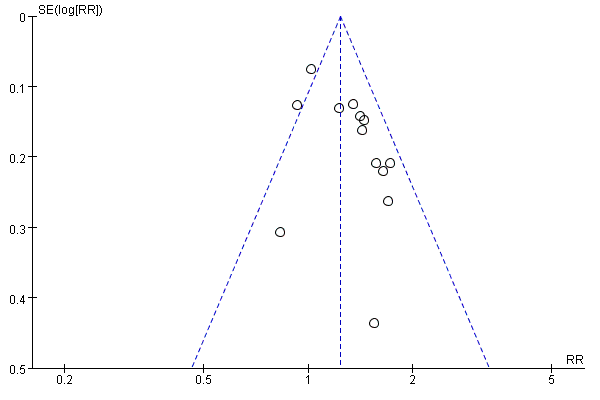


**Figure S2a. Tests for publication bias for RR of clinical response by Funnel plot of clinical response.**


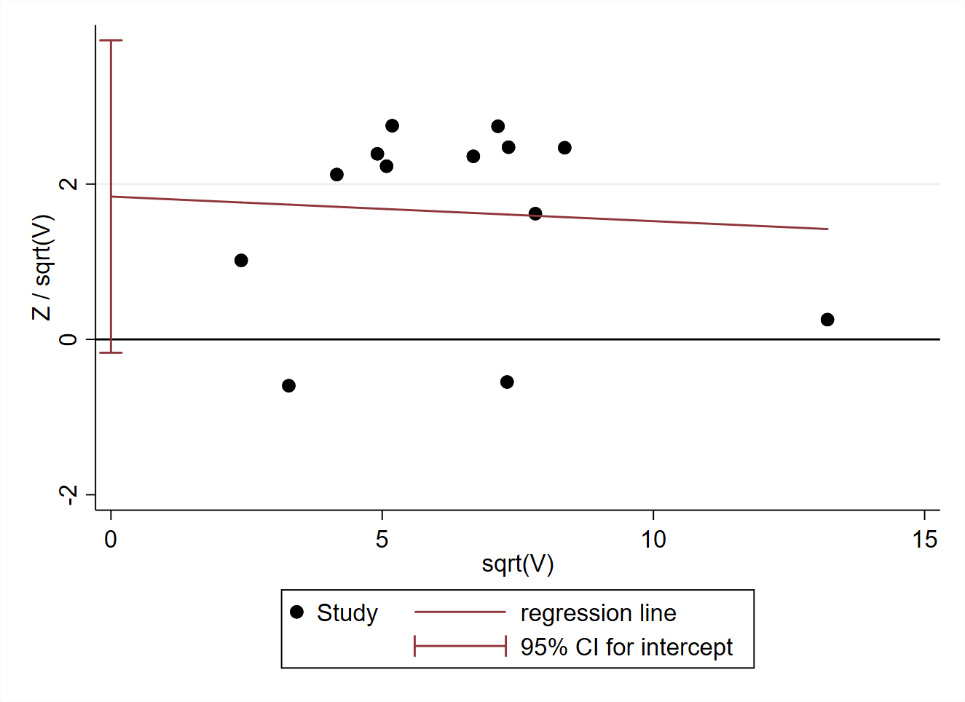


**Figure S2b.Tests for publication bias for RR of clinical response by adjusted Galbraith(P=0.069).**


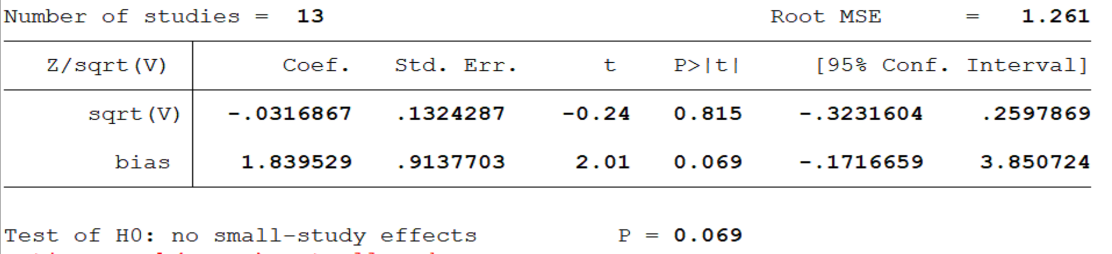


**Appendix 9 Forest plots of the effects of Amikacin nebulization on the secondary outcomes**


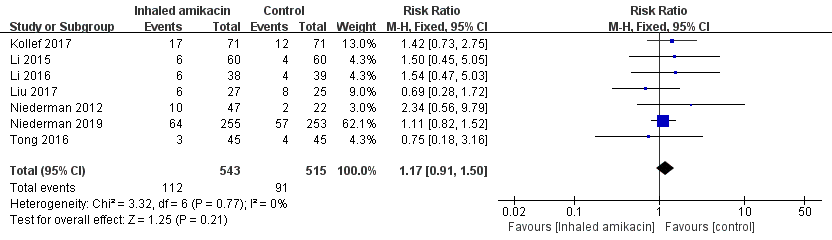


Figure S3. Forest plots of the effects of aerosolized amikacin on overall mortality.


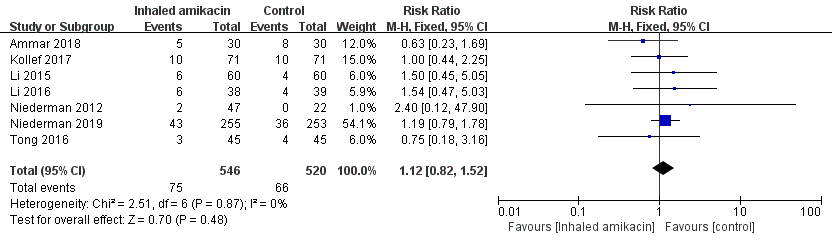


Figure S4. Forest plots of the effects of aerosolized amikacin on pneumonia associated mortality.


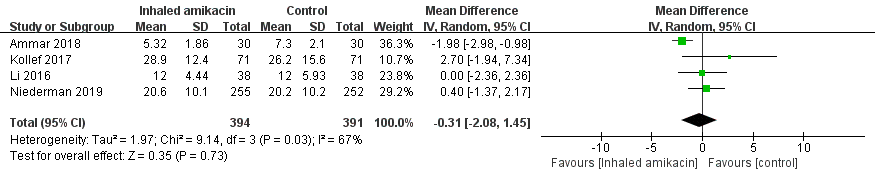


Figure S5. Forest plots of the effects of aerosolized amikacin on the length of stay in intensive care unit.


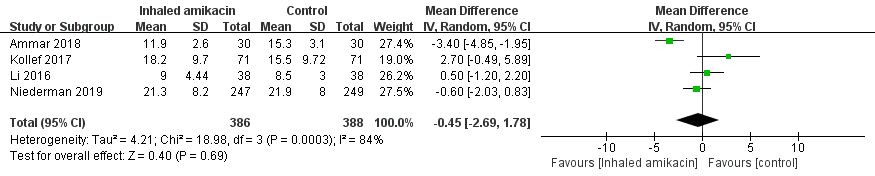


Figure S6. Forest plots of the effects of aerosolized amikacin on duration of mechanical ventilation.


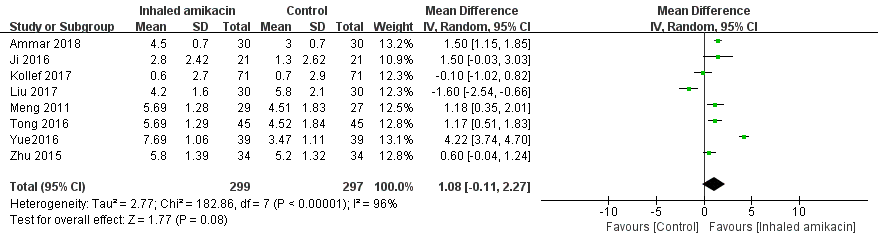


Figure S7. Forest plots of the effects of aerosolized amikacin on ∆ clinical pulmonary infection score.


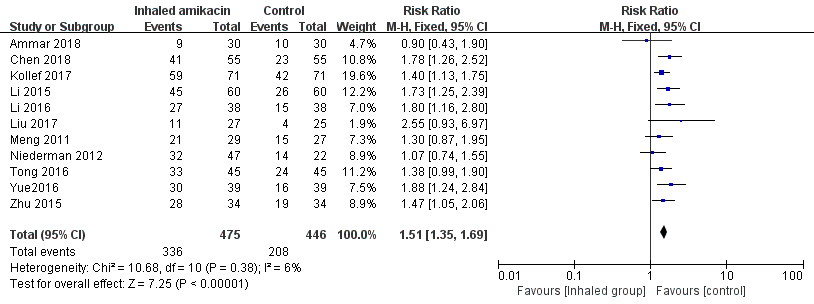


Figure S8. Forest plots of the effects of aerosolized amikacin on microbiologic eradication.


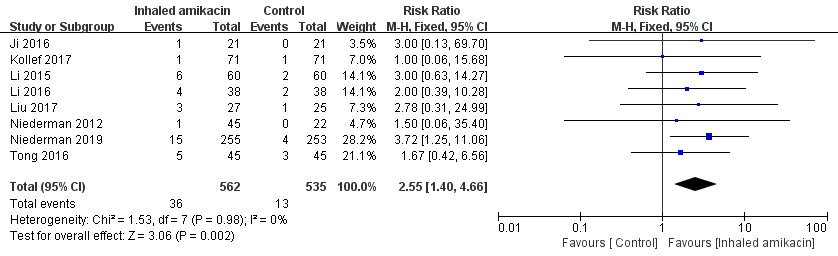


Figure S9. Forest plots of the effects of aerosolized amikacin on bronchospasm.


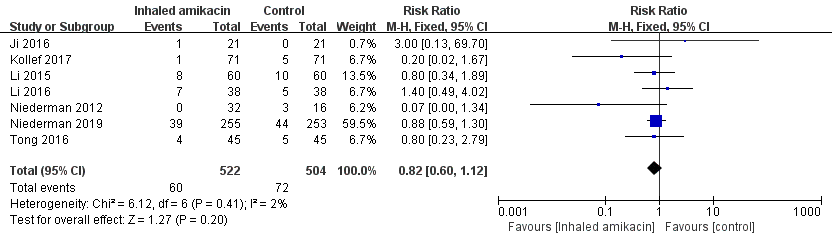
Figure S10. Forest plots of the effects of aerosolized amikacin on nephrotoxicity.
